# Kerion as an atypical presentation of tinea capitis in an elderly patient: A case report

**DOI:** 10.1016/j.jdcr.2025.11.018

**Published:** 2025-12-04

**Authors:** Mihir M. Shah, Karan S. Cheema, Alina S. Feng, Kerri E. Rieger, Gordon H. Bae

**Affiliations:** a Stanford University School of Medicine, Stanford, California; b School of Public Health, Brown University, Providence, Rhode Island; c Department of Dermatology, University of California San Francisco, San Francisco, California; d Department of Dermatology, Stanford University School of Medicine, Redwood City, California

**Keywords:** hair loss, kerion, minoxidil, tinea capitis

## Introduction

Kerion is the inflammatory extreme of tinea capitis, a dermatophyte infection typically seen in children but increasingly reported in adults, particularly the elderly.^1^ Clinical presentation in older adults varies and may mimic bacterial infections or inflammatory conditions, leading to possible delays in appropriate diagnoses and treatments.^2^^,^^3^ Globally, *Microsporum canis* and *Trichophyton tonsurans* are among the most common etiologic agents, representative of zoophilic and anthropophilic transmission patterns, respectively; *M. canis* is most prevalent among school-aged children within Europe and parts of Asia, whereas *T. tonsurans* is more common in North America or Africa.^4^ In elderly populations, comorbidities, atypical morphology, and lower clinical suspicion may further complicate recognition.^2^ Adult manifestations of kerion range from patchy hair loss to more significant alopecia typically derived from the onset of painful masses.^2^ We present a case of kerion in an 81-year-old female, highlighting the diagnostic and therapeutic challenges observed in an atypical kerion demographic.

## Case report

**Fig 1:**
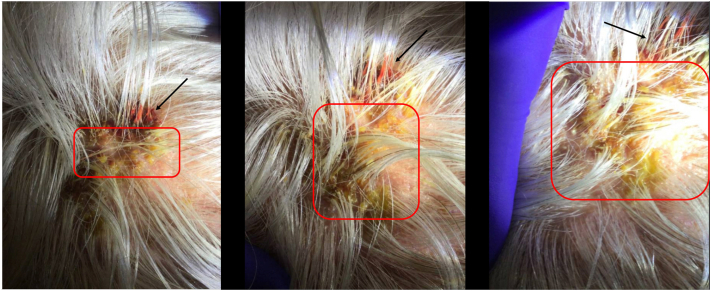
Initial images associated with consultation to dermatology and date of wound culture. The *red boxes* represent areas of crusting. The *black arrows* represent areas of edema/erythema.

An 81-year-old healthy woman with no significant medical history or medication use presented to express care with a 2-week history of painful, eroded plaques on the scalp with purulent discharge, honey-colored and hemorrhagic crusting, and subjective fevers. Examination revealed tender lesions with surrounding erythema and edema (Fig 1). Initial empiric treatment with oral trimethoprim-sulfamethoxazole was prescribed for suspected bacterial impetiginization. Despite adherence, her condition worsened, prompting urgent dermatologic referral.

Dermatologic examination showed multiple coalescing boggy plaques across the parietal and occipital scalp, with extensive crusting, pustules, and areas of alopecia. Regional lymphadenopathy was absent.

**Fig 2:**
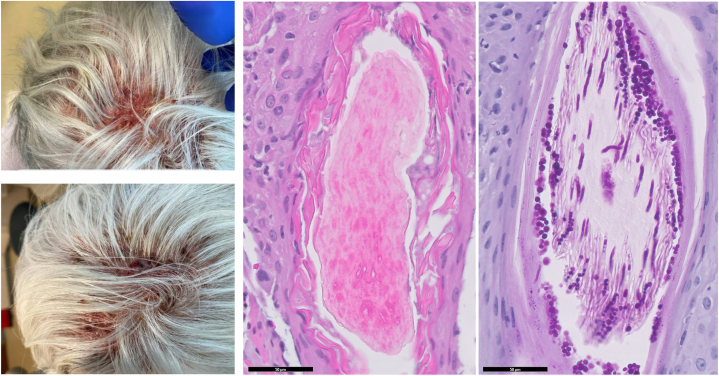
Scalp images and punch biopsy histology from follow-up visit. Fungal hyphae and spore forms are seen within the hair follicle, within and surrounding the hair shaft. (left panel, H&E; right panel, PASd stain; 50 μm scale bar, original magnification (400×)

Punch biopsies revealed fungal hyphae and spore forms both within and surrounding the hair shaft, consistent with mixed endothrix and ectothrix dermatophytosis. Periodic acid–Schiff staining highlighted abundant fungal elements (Fig 2), and tissue culture grew >100 colonies of Trichophyton species, confirming a dermatophytic etiology.

The patient was initiated on oral terbinafine 250 mg daily for 8 weeks, supplemented with topical ketoconazole 2% shampoo daily and dilute bleach soaks twice daily to assist in debridement of thick crusts. Clinical improvement was observed, with improvement in pain within 1 week and near resolution of symptoms within 4 weeks. By 4 weeks, about half of the crusting had resolved, with near-complete clearance by 8 weeks except for minimal residual encrustation and superficial erosions. However, residual patches of alopecia persisted, affecting approximately 50% of the scalp surface.

**Fig 3:**
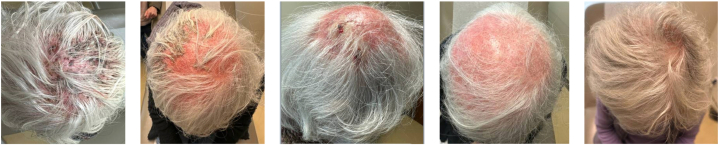
Progression of treatment from the start of terbinafine (left); Week 4 of 8-week course (second image); After completion of 8-week course (third image); Start of minoxidil (fourth image); 2-3 weeks after stopping minoxidil (right).

Topical minoxidil 5% foam was initiated 1 month after completing terbinafine, resulting in near-complete hair regrowth after 5 months. (Fig 3).

## Discussion

Kerion in elderly patients presents a diagnostic dilemma in which clinical appearance often mimics bacterial or inflammatory scalp conditions. The marked inflammation, purulent discharge, hemorrhagic crusting, and tenderness observed in our patient are more typical of bacterial infections, leading to empiric antibiotic treatment and delayed antifungal therapy. This diagnostic delay is well-described in literature, with 1 study showing an average of 22.5 weeks to diagnosis.^5^ When scalp lesions do not respond to standard antibacterial regimens, dermatophytosis should be considered early, and diagnostic testing should be pursued. Biopsy and culture currently remain gold standards for establishing a kerion diagnosis, particularly in adults where the index of suspicion is lower.^2^^,^^6^^,^^7^

Histopathology in this case revealed fungal hyphae and spores both within and surrounding the hair shaft. These findings are consistent with a mixed endothrix and ectothrix dermatophytosis, and they confirm active invasion of the follicle, which explains the patient’s significant inflammatory response. Mixed patterns may also correlate with greater follicular destruction, thereby increasing the risk of alopecia.^8^ These histologic insights, when combined with positive culture growth of the Trichophyton species, provides strong evidence for kerion and justifies the need for systemic antifungal therapy.

Systemic antifungal therapy is the cornerstone of kerion management. Terbinafine, used in this case, is often preferred for its fungicidal activity against *Trichophyton* species and its ability to concentrate within the hair shaft.^9^ In elderly patients, antifungal selection requires careful consideration of hepatic function and potential drug–drug interactions. Alternative agents, such as itraconazole or griseofulvin, may be preferred for Microsporum infections; however, terbinafine is favored for Trichophyton-related kerion, as it requires a shorter treatment course and has a better safety profile.^9^^,^^10^ Adjunctive measures such as topical ketoconazole shampoo and dilute bleach soaks may help reduce surface fungal burden, enhance scalp hygiene, or debride the crusting.^11^^,^^12^ When used synergistically, these strategies may allow for gradual clinical improvements and possible resolution of active infection.

Alopecia is one of the most challenging sequelae of kerion, often resulting in lasting cosmetic and psychosocial impacts. Inflammatory destruction of the follicle due to kerion can likely result in cicatricial alopecia if untreated; this has been previously shown to present in 56.1% of patients.^12^ Furthermore, even with treatment, hair regrowth may remain incomplete. Our patient was left with significant patches of hair loss despite clearance of infection after 8 weeks of systemic therapy. However, with our case in mind, there is a likelihood that some hair loss may be due to a telogen effluvium-like process. Specifically, the addition of topical minoxidil 5% foam promoted near-complete hair regrowth within 5 months. Topical minoxidil 5% has demonstrated efficacy in promoting hair regrowth in non-androgenetic alopecia, including alopecia areata and post-inflammatory hair loss, supporting its use as a therapeutic adjunct in this case.^13^ Minoxidil stimulates follicular activity and prolongs anagen phase, which may aid recovery of follicles not irreversibly damaged by inflammation.^14^ Its success here suggests clinicians could consider minoxidil in similar contexts to optimize cosmetic outcomes and improve quality of life. However, the findings from a single case report are limited in their generalizability, and further studies are needed to confirm the efficacy of minoxidil in post-kerion alopecia, especially as there could be spontaneous hair regrowth months after infection.

The incidence of adult and elderly tinea capitis is rising globally, with geographic variation in causative species.^4^^,^^6^^,^^7^ As populations age and the use of immunosuppressive therapies increases, dermatologists may encounter more cases of fungal scalp infections in older adults, although no direct link has been established. Awareness of shifting epidemiology is critical for clinicians, as reliance on pediatric-focused clinical indications may not serve to be directly translatable. Diagnostic protocols traditionally geared toward children should be adapted to include adult-specific considerations, especially in elderly patients with atypical presentations as shown here.

Ultimately, the management of kerion in elderly patients is multidimensional and it not only involves the eradication of fungal infection but also attention to the cosmetic and psychological impact of alopecia. Integrating systemic antifungals, adjunctive local therapies, and hair regrowth agents can optimize both medical outcomes and patient satisfaction.

## Conclusion

This case underscores kerion as an important diagnostic challenge in the elderly. We found that early recognition and treatment of kerion with systemic terbinafine, supported by topical therapy and minoxidil for post-infectious alopecia, facilitated near-complete recovery. As the global population rise and the number of elderly people increases, dermatologists should maintain increased awareness of adult and elderly tinea capitis to ensure timely diagnosis and treatment.

## Conflicts of interest

None disclosed.
